# Experience and Outcomes of Primary Percutaneous Coronary Intervention in a Tertiary Care Hospital in South Punjab, Pakistan

**DOI:** 10.7759/cureus.50024

**Published:** 2023-12-06

**Authors:** Ammar Akhtar, Muhammad S Saleemi, Qazi M Zarlish, Muhammad B Arshad, Kashif A Hashmi, Hamza Ghafoor

**Affiliations:** 1 Cardiology, Chaudhary Pervaiz Elahi Institute of Cardiology, Multan, PAK; 2 Cardiology, Nishtar Medical University, Multan, PAK; 3 Cardiology, Chaudhry Pervaiz Elahi Institute of Cardiology, Multan, PAK

**Keywords:** timi flow, door-to-balloon time, total ischemic time, st-elevation myocardial infarction, primary percutaneous coronary intervention

## Abstract

Background: Primary percutaneous coronary intervention (PCI) is the emergency procedure and gold standard for the treatment of ST-Elevation Myocardial Infarction (STEMI).

Objective: To share the experience and outcomes of primary PCI in a tertiary care hospital in South Punjab, Pakistan.

Methods: A descriptive cross-sectional study was planned from the 1^st^ of April, 2023 to the 30^th^ of September, 2023. All patients presenting with acute STEMI undergoing primary PCI were included in the study. Data regarding patient demographics, risk factors, procedural characteristics, time variables, and in-hospital events was observed.

Results: A total of 1132 patients were included in the study. There were 934 (82.5%) males. Smoking is the most common risk factor among all the patients. Anterior wall myocardial infarction is the most common STEMI and the left anterior descending artery is the culprit vessel in 58.3% (n=660) of patients. Single vessel disease was present in 34.6% (n=392) of the patients. Thrombolysis in Myocardial Infarction (TIMI )Flow III was achieved in 80% (n=906) of patients after primary PCI. The average total ischemic time of the patients included in the study was 5 hours and 48 minutes, with a standard deviation of 3 hours and 55 minutes. Our mortality was 3.5% (n=40).

Conclusion: Our patients take a longer time to present to the PCI-capable centers. Despite their late presentation, primary PCI has better outcomes for treating acute STEMI.

## Introduction

Acute cardiovascular events, if not treated promptly, may prove fatal. One such harrowing class is the ST-segment elevation myocardial infarction (STEMI), an alarming subset of heart attack that strikes abruptly and demands immediate medical attention [1]. Every moment that ticks by after a STEMI commences equates to cardiac tissue death, and making timely interventions may ensure survival and recovery. Primary Percutaneous Coronary Intervention (PCI) stands out as a beacon of hope for these patients who are experiencing acute myocardial infarction.

Historically, thrombolytic therapy was the gold standard for managing acute STEMI patients, a treatment reminiscent of days when the understanding of cardiovascular science needed much improvement [2]. But as medical technology and knowledge advanced, so did the evolution of treatments. Fast forward to today, and we see primary PCI championed as the primary reperfusion strategy. As affirmed by Ibanez and colleagues in their 2018 guidelines, primary PCI has demonstrated superior outcomes in comparison to thrombolytic therapy, enhancing not just survival rates but also the quality of life post-STEMI [1].

However, like all medical interventions, its success orbits around timeliness and expertise, which brings forth the question: How effectively is this procedure being implemented worldwide? Our world, despite being more interconnected than ever, is still struggling with healthcare disparities. And when it comes to critical procedures like primary PCI, the impact of these disparities becomes distressingly evident. Cardiovascular diseases remain the grim reaper, In the U.S., cardiovascular disease claims a life roughly every 33 seconds. In 2021 alone, heart disease accounted for about 695,000 fatalities, making up one in every five deaths [2]. A significant chunk of this statistic is represented by ischemic heart disease, and within this, STEMI cases take the fore. In Pakistan, 29.1% of deaths were reported due to ischemic heart disease in 2017 [3].

The prevalence of cardiovascular disease in Pakistan is reported to be one in four in men and one in three in women above 40 years of age [3]. Though Europe has made commendable strides with a reported increase in PCI centers, the scenario is not the same universally [4]. Garceau et al. echoed concerns about treatment delays leading to a surge in mortality rates in certain regions [5]. Such delays and, sometimes, complete unavailability of primary PCI in certain areas underscore the need for a comprehensive evaluation. Thus, we embark on this research voyage with a clear objective to evaluate the real-world experiences and outcomes of primary PCI in acute STEMI patients in a tertiary hospital in Punjab, Pakistan. Our research is directed by the numerous questions that hover around this topic. Are patients getting timely access to PCI? What kind of outcomes are we observing across diverse populations and geographies? Are there specific complications or challenges unique to primary PCI that need more attention?

We are diving into this study because we have some big questions. While a lot has been written about how good primary PCI is, there's less out there about what patients really experience in our setup and how they fare afterward. We want to fill in those gaps. By doing so, we hope to give our doctors’ community a better idea of where we lag and how we can improve our system. In simple terms, with all the amazing medical breakthroughs happening today, it is important to check how they are working in a developing world. Our research is a small step in that direction, hoping to shed more light on how primary PCI works for those with acute STEMI in tertiary hospitals in South Punjab, Pakistan.

## Materials and methods

This descriptive cross-sectional study was carried out in the Emergency Department along with the Department of Interventional Cardiology at Chaudhary Pervaiz Elahi Institute of Cardiology, Multan, for six months from the 1st of April, 2023, to the 30th of September, 2023. The study was conducted after getting permission from the institutional Ethical Review Board. Informed consent was taken from all the subjects. Patients presenting with acute STEMI of both genders and all age groups were included in the study. Patients who did not give informed consent and who had a history of coronary artery bypass grafting and chronic kidney disease were excluded from the study. All patients who were admitted to the emergency department of our hospital with retrosternal central crushing chest pain of severe intensity, chest heaviness, or chest tightness of squeezing and gripping in character radiating towards the left arm, neck, or jaws exacerbating with exertion had undergone standard 12-lead electrocardiography (ECG) in the resuscitation bay of our emergency. The ECG will be considered as indicating a new ST elevation MI if there is a noticeable elevation of the ST-segment at the J-point in chest leads V2 and V3, measuring 2 mm or more in males, 1.5 mm or more in females, and 1 mm or more in 2 or more contagious leads.

The patient was immediately discussed in the catheterization laboratory for primary PCI. The diagnosis was explained to the family and then informed consent was taken to shift the patient to the Catheterization laboratory. All the patients were given chewable tablet aspirin 300 mg and tablet clopidogrel 600 mg before shifting to primary PCI. Meanwhile, blood samples for complete blood workup, renal parameters, random blood glucose level, HbA1C, and lipid profile were also withdrawn and sent to the pathology laboratory. The primary PCI was performed by a senior cardiologist with at least three years of post-fellowship experience. After primary PCI, the patient was shifted to the coronary care unit. After stabilizing the patient, a detailed history was taken from the patient. Patients were asked about the duration between the onset of symptoms and arrival at our hospital to calculate total ischemic time. Prior history of myocardial infarction, coronary angiography, and PCI was also taken and the record was also reviewed, if available with the patient. History regarding conventional risk factors of coronary artery disease like diabetes, hypertension, dyslipidemia, smoking, and family history was also gathered. Door-to-balloon time was also calculated from hospital records. If the patient has a previous history of PCI then the duration of PCI was also asked to assess for acute, subacute, and late stent thrombosis. Pre-procedural complications of MI like cardiac arrest, any sort of tachyarrhythmias or bradyarrhythmia, hypotension, shock, and pulmonary edema were also noted from the hospital record. Coronary angiography and primary PCI were reported by the same operator who performed the procedure. Use of glycoprotein IIb/IIIa inhibitor tirofiban and thrombectomy catheter, if done during the procedure, was also noted. The procedure result was also noted as Thrombolysis in Myocardial Infarction (TIMI) flow in the infarct-related artery. Forty-eight hours after the procedure, the left ventricle (LV) ejection fraction calculated by the Biplane Simpson method was used to assess the left ventricular systolic function. Post-procedure complications related to MI as well as related to procedure and access site complications were also noted. All the data was calculated on a predesigned proforma.

Statistical analysis

Computer software SPSS, version 23.0 (IBM Corp., Armonk, NY) was used to enter and analyze the data. Quantitative variables like age, total ischemic time, door-to-balloon time, etc., were represented by mean and standard deviation. Categorical variables like conventional risk factors of coronary artery disease, use of glycoprotein IIb/IIIa inhibitor, number of vessel involvement, pre- and post-procedure complications, assess site complications, and TIMI flow in infarct-related arteries, etc., were represented by frequency and percentage. The relation of ejection fraction and TIMI flow in the infarct-related artery with total ischemic time was also tested by the Chi-square test. A p-value of less than 0.005 was considered significant.

## Results

A total of 1132 patients were included in the study. Among these, 934 (82.5%) were males and 198(17.5%) were females. The mean age of the patients was 53.51±11.37 years. The youngest patient included in the study was 18 years and the oldest patient was 86 years. One hundred and forty (10.6%) patients were less than 40 years of age, 858 (75.8%) were of age group 40 to 65 years, and 154 (13.6%) were older than 65 years. Three hundred and forty-eight (30.7%) were diabetics, 324 (28.6%) were hypertensives, 342 (30.2%) had dyslipidemia, 200 (17.7%) had a family history of ischemic heart disease, and 652 (57.6%) were smokers. Eighty-six (7.6%) patients had a previous history of myocardial infarction, eight (0.7%) had a history of primary PCI and 12 (1.1%) had a history of elective PCI in the past. Thirty (2.7%) patients had STEMI due to stent thrombosis. Out of these 30, six (20%) patients had acute stent thrombosis (within seven days of PCI), 14 (46.7%) had subacute stent thrombosis (after eight days but within one month of PCI), and 10 (33.3%) had very late stent thrombosis, i.e., after one year of PCI. The average total ischemic time of the patients included in the study was 5 hours and 48 minutes, with a standard deviation of 3 hours and 55 minutes. The minimum total ischemic time was 45 minutes and the maximum total ischemic time was 23 hours and 30 minutes. One hundred and eighteen (10.4%) patients have a total ischemic time of less than two hours, 618 (54.6%) have ischemic time of two to six hours, 320 (28.3%) patients have ischemic time of six to 12 hours and 26 (6.7%) had ischemic time of 12 to 24 hours. Table 1 shows the frequency and percentages of different types of STEMI and infarct-related arteries.

**Table 1:** Types of ST-elevation myocardial infarction treated with primary percutaneous coronary intervention and infarct-related arteries

Type of STEMI	Frequency	Percentage
Extensive Anterolateral wall Myocardial infarction	56	4.95%
Anterior wall Myocardial infarction	606	53.5%
High lateral wall Myocardial infarction	10	0.9%
Inferolateral wall Myocardial infarction	18	1.6%
Infero-posterolateral wall Myocardial infarction	16	1.4%
Infero- posterior wall Myocardial infarction	28	2.5%
Inferior wall Myocardial infarction	218	19.3%
Inferior wall Myocardial infarction with Right ventricular infarct	178	15.7%
Posterior wall Myocardial infarction	2	0.2%
Infarct-Related Arteries		
Left circumflex artery	90	8.0%
Left anterior descending artery	660	58.3%
Obtuse marginal artery	6	0.5%
Right coronary artery	372	32.9%
Posterior left ventricular artery	2	0.2%
Number of vessels involved		
Single vessel disease	392	34.6%
Two vessel disease	362	32.0%
Three vessel disease	344	30.4%
Left main coronary artery disease	32	2.8%
Normal coronaries	2	0.2%

Out of 1132 patients shifted for primary PCI, 984 (86.9%) patients were treated with primary PCI and 148 (13.1%) patients were deferred from Primary PCI after coronary angiography. The different reasons for deferring patients from primary PCI are three-vessel coronary artery disease in 78 (6.9%) cases, significant Left main coronary artery disease along three-vessel disease in 12 (1.1%) patients, diffuse coronary artery disease in four (0.4%) patients, heavy clot burden in 16 (1.4%) patients, distal posterior left ventricular (PLV) branch in two (0.2%) patients, recanalized vessel in 28 (2.5%) patients with TIMI III flow, mile disease in two (0.2%) patients, normal coronaries in two (0.2%) patients and difficult engagement of infarct-related artery in four (0.4%) patients. Out of these 148 patients, 106 patients had been treated by thrombolysis with streptokinase. The average door-to-balloon time of the patients included in the study was 41 minutes, with a standard deviation of 25 minutes. The minimum door-to-balloon time was 16 minutes, and the maximum door-to-balloon time was 135 minutes. The right femoral artery was used to do primary PCI in 600 (53%) patients, and the right radial route was used in 532 (47%) patients. Table 2 shows the details of primary PCI done in the remaining patients and the results of primary PCI in terms of TIMI flow in the infarct-related artery.

**Table 2:** Detail of primary PCI and results of primary PCI TIMI: Thrombolysis in myocardial infarction; PCI: Percutaneous coronary intervention.

Strategy of Primary PCI	Frequency	Percentage
Pre-dilation followed by stent placement and post-dilation	874	77.2%
Direct stent placement with no pre-dilation and post-stent dilation	42	3.7%
Only plain old balloon angioplasty	70	6.2%
Use of Glycoprotein IIb/IIIa inhibitor (tirofiban)	932	82.3%
Use of thrombectomy catheter	42	3.71%
Results of Primary Percutaneous Coronary Intervention in terms of TIMI Flow		
TIMI Flow 0	20	1.8%
TIMI Flow I	12	1.1%
TIMI Flow II	46	4.1%
TIMI Flow III	906	80.0%

**Figure 1:**
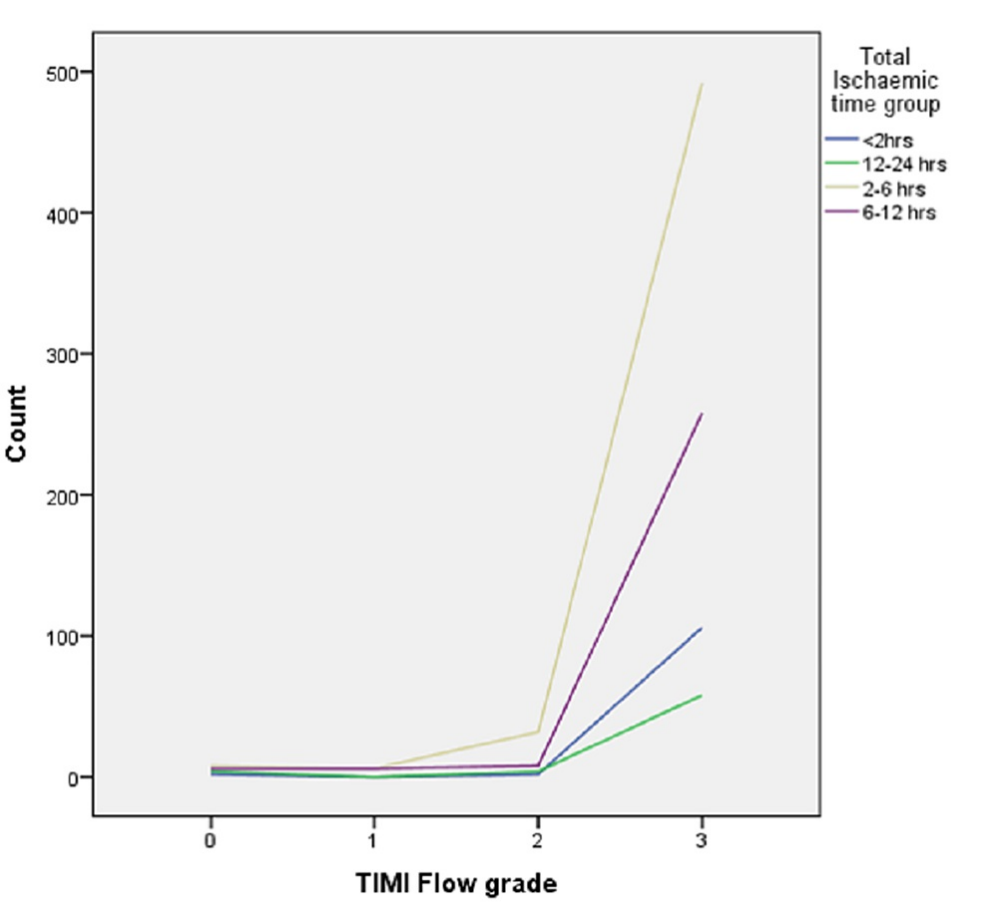
Graph between TIMI flow in infarct-related artery against total ischemic time TIMI: Thrombolysis in myocardial infarction.

Figure 1 shows the association of TIMI flow in infarct-related arteries with the total ischemic time representing that less ischemic time means better TIMI flow. The p-value came out to be 0.001.

**Figure 2:**
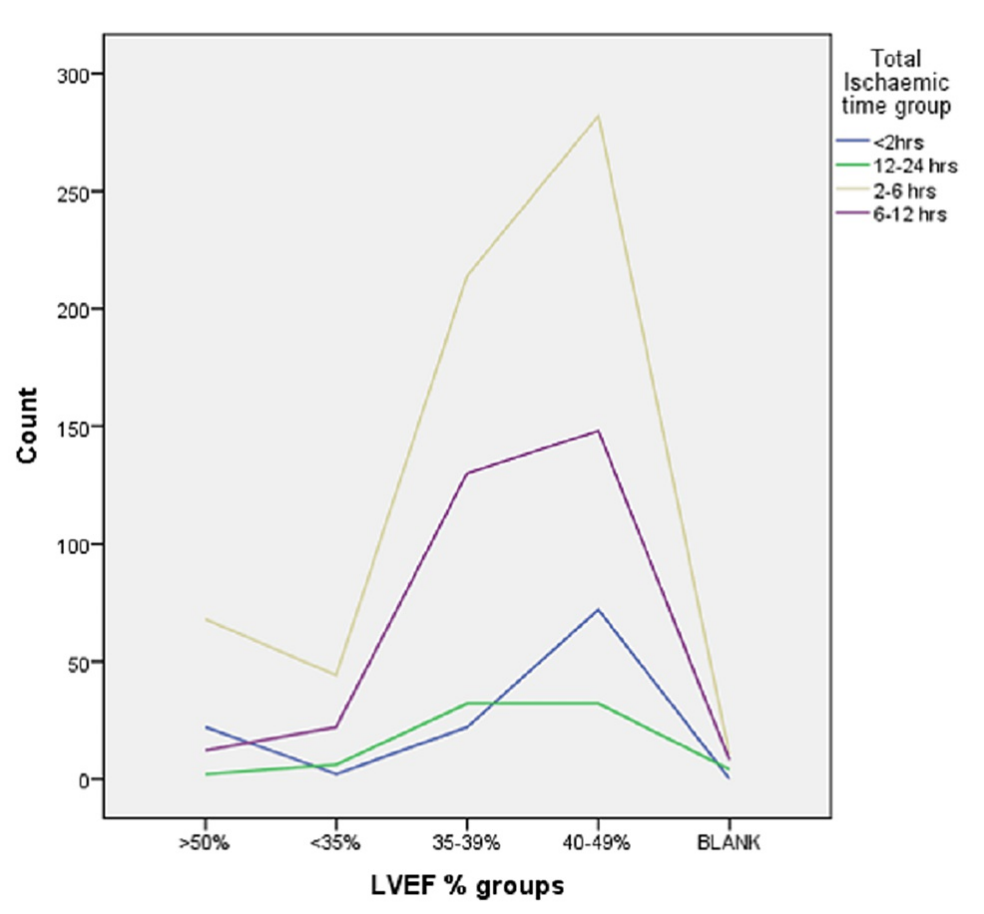
Graphical presentation showing the effect of total ischemic time on ejection fraction LVEF: Left ventricular ejection fraction.

Transthoracic echocardiography was done to assess the left ventricular (LV) systolic function, which was assessed by calculating ejection fraction (EF) via the Biplane Simpson method. Seventy-four (6.5%) patients have ejection fraction (EF) less than 35%, 398 (35.2%) have EF 35-39%, 534 (47.2%) have EF 40-49% and 104 (9.2%) have EF more than 50%. We were unable to calculate EF in 22 patients due to mortality within 24 hours. Figure 2 shows the graph between EF against the total ischemic time which shows that early presentation is beneficial in preserving the LV function. The p-value came out to be 0.0001.

Different complications have been observed during or after primary PCI. Cardiac arrest happened in 60 (5.3%) patients. Heart failure of Killip Class II occurred in 128 (11.3%), Killip Class III in 6 (0.5%), and Killip Class IV in 32 (2.8%) patients. Twenty-eight (2.5%) patients had ventricular tachycardias, 18(1.6%) had atrial fibrillation and 34 (3.0%) had bradycardias. Acute kidney injury was developed in 20 (1.8%) patients, and two (0.2%) patients had stroke. Four (0.4%) patients had access site complications. Two patients (0.2%) have retroperitoneal hematomas, one (0.1%) has a superficial hematoma at the puncture site of the femoral artery and one (0.1%) has a hematoma at the radial artery puncture site. Forty (3.5%) patients were unable to survive. The most common cause of mortality in our setup was heart failure in 26 (2.3%) patients, followed by sudden cardiac arrest due to arrhythmic complications in 12 patients (1.06%), and two patients (0.2%) died due to stroke.

## Discussion

In this study, we have aimed to show our experience and outcomes of primary PCI in our setup, which is situated in a developing country with limited resources. We have analyzed demographics, angiographic findings, procedural results, and complications during and after the procedure. Different randomized trials have proven the efficacy of Primary PCI over fibrinolysis [6]. The European and American guidelines have recommended primary PCI as the gold standard for the treatment of STEMI [7]. The demographics of our study showed that the males are predominantly more presenting with acute STEMI as compared to the females, which is similar to the study conducted in India by Qamar et al., which reported 16% of patients to be females presenting with STEMI [8]. One alarming situation in our study was that 10.6% of patients were aged less than 40 years which is similar to the study conducted by Qamar et al. in India [8]. This shows that young people also have an increasing risk of coronary artery disease day by day.

Our study showed that smoking is the most common risk factor among patients presenting with acute STEMI. These results are also comparable to the results of a study conducted by Jahic in Bosnia [9]. The average total ischemic time in our study was 5 hours and 48 minutes (348 minutes), which is more than the total ischemic time reported by Jahic (193±118.2 minutes) in Bosnia [9] but comparable to the total ischemic time in a similar study conducted in India by Durgaprasad et al. which is 369 minutes [10]. This difference between the developing and developed world is due to the poor ambulance services and poor transportation services causing delays in the arrival of patients at PCI-capable hospitals.

The average door-to-balloon time in our study was 41 minutes which is less than the door-to-balloon time observed by Durgaprasad et al. in India, which was reported to be 58 minutes [10]. Door-to-balloon was reported to be 67 min in 2019 in New Zealand by Hansen et al. [11]. The difference in the average door-to-balloon time shows that our setup has better in-hospital services and a more efficient system to activate the catheterization laboratory for primary PCI.

The in-hospital mortality varies between 6% and 14% in literature from the national registries of the 32 European countries [12]. The mortality is affected by multiple factors, among them: age, Killip class, time delay to treatment, history of prior myocardial infarction, diabetes mellitus, etc. In-hospital mortality in our study was 3.5% and the most common cause of mortality is heart failure, which is due to damage of functioning myocardium leading to pump failure. In this situation, the patient usually needs circulatory support like an intra-aortic balloon pump or impella devices, which are unavailable in our setup. Despite this, our mortality is comparable to a study conducted by Jahic E, who reported 3.1% mortality [9], and Durgaprasad et al., who reported 3.6% mortality in their study [10].

We have seen that anterior wall myocardial infarction is the most common myocardial infarction presenting in an emergency, and the left anterior descending artery is the most commonly involved vessel. About 34.6% of the patients have single-vessel disease. Similar results have been reported by Mujtaba et al. in their study conducted in Sindh, Pakistan [13].

The no-reflow phenomenon is still a dreadful challenge not only for the STEMI patients but also for the operator. It is an independent poor prognostic factor in addition to the other risk factors. In the study conducted in India by Datta G, no flow was reported in 7.75% of patients [14]. Our results of TIMI Flow grade 0 and I were similar to the reported incidence in the literature. Out of the total, 80% of our patients have achieved TIMI III flow which is comparable to similar studies done previously [9]. We have implanted stents in infarct-related arteries with pre-dilation and post-dilation in 77.2% and plain old balloon angioplasty was done in 6.2% of patients. Literature has shown comparable results to our study [9] which shows that our setup has done work comparable to the other centers in the developed world. We have used thrombectomy catheters in 3.71% of patients with heavy clot burden as a bailout procedure. Studies have shown the use of thrombus aspiration up to 29% as a bailout procedure leading to more successful PCIs [15]. The less use of thrombectomy catheters is due to the non-availability of the advanced instruments in our setup. Rather we have higher use of Glycoprotein IIb/IIIa inhibitor as it is easily available as compared to similar studies [9].

We have reported that the primary PCI is beneficial in preserving LV systolic function. Only 6.5% of patients have an ejection fraction of less than 35%, indicating severe LV systolic dysfunction. An increase in the total ischemic time also increases the risk of LV systolic dysfunction, which is clearly shown in the results. These results are also similar to the results reported in the literature [16,17].

Our study has a few limitations. First, it has a limited number of patients and a single-center study. The second limitation is the lack of follow-up with the patients regarding further planning and management of non-infarct related arteries and long-term cardiovascular events. For this, it is the need of an hour to conduct a study involving multiple centers in the country and with a proper follow-up of the patients, which will be done in future studies.

## Conclusions

Primary percutaneous coronary intervention is not only myocardium-saving but also a life-saving procedure in patients with ST- Elevation Myocardial Infarction. Males are predominantly suffering more from this disease. Smoking is the most common risk factor. Anterior wall MI is the most commonly presented STEMI. The left anterior descending artery is the most commonly involved infarct-related artery. In the case of STEMI, time is precious. Less total ischemic time means less myocardium damage.
